# Exploiting the Applicative Potential of Hydroxyethyl Cellulose-Based Composites by Composition-Tailoring of the Optical and Dielectric Features

**DOI:** 10.3390/polym17172315

**Published:** 2025-08-27

**Authors:** Andreea Irina Barzic, Iuliana Stoica, Raluca Marinica Albu, Igori Belotercovschii, Victor Suman, Victor V. Zalamai, Victor Cojocaru

**Affiliations:** 1 “Petru Poni” Institute of Macromolecular Chemistry, 41a Grigore Ghica Voda Alley, 700487 Iasi, Romania; 2 “D. Ghiţu” Institute of Electronic Engineering and Nanotechnologies, Technical University of Moldova, 2028 Chisinau, Moldova; 3 National Center for Materials Study and Testing, Technical University of Moldova, 2004 Chisinau, Moldova

**Keywords:** cellulose ether, inorganic fillers, morphology, refractive index, electrical capacity

## Abstract

This work deals with the preparation of a novel set of ternary polymer composites, where the matrix is a cellulose ether and the reinforcement agent is a 50:50 mixture of TiO_2_ nanoparticles with PbCl_2_ micropowder (0.25–4 wt%). The attained film samples are investigated from morphological, optical, and electrical points of view to explore the applicative potential as LED encapsulants or flexible dielectric layers for capacitors. Morphological analyses at micro- and nanoscale evidence the level of distribution of the fillers blended within the matrix. UV-VIS spectroscopy and refractometry emphasize that at 0.5 wt% the samples display the best balance between transparency and high refractive index, which matches the applicative criteria for LED encapsulation. The electrical testing with broadband dielectric spectrometer proves that the dielectric constant at 1 kHz of the composite with 4 wt% fillers is enhanced by about 6.63 times in comparison to the neat polymer. This is beneficial for designing eco-friendly and flexible dielectrics for capacitor devices.

## 1. Introduction

Many technologies have evolved over the past few years as a result of the scientific community’s ongoing efforts of producing novel materials, analysis techniques or new processes [1]. The driving factor for materials development is closely linked to the applicative purpose, so the current library of materials contains various synthetic compounds with well-established properties of great utility in industrial sectors. More and more studies are proving that combination of polymers with inorganic compounds represents a facile and relatively low-cost approach of achieving composite materials with refined chemical and physical performance [2,3]. In the context of plastic pollution, there is a growing tendency of studying biodegradable polymers that are soluble in non-toxic solvents, such as cellulose derivatives [4]. Aside from the low impact on the environment, these cellulose ethers display several advantages, such as facile renewability, processability into films/fibers/gels, water solubility, enhanced adhesion, mechanical resistance, and high optical transparency [5,6]. Hydroxyethyl cellulose (HEC) is a nonionic cellulose ether, which is known for its stability under variable pH, thermal resistance up to 200 °C, excellent film-forming ability, high insulation, and good optical properties [7]. Loading HEC with inorganic particles is a suitable route towards novel materials with tailored properties [8,9,10]. Depending on the characteristics of the incorporated filler, the properties of HEC can be shifted to match the practical criteria, which may include antibacterial activity [11], conductive characteristics [12], high electrical permittivity [13,14,15], or high refraction features [14]. Therefore, HEC composites are versatile as a result of combination of materials with distinct properties that render unique performance that cannot be reached with a single material.

HEC presents many interesting optical properties, like UV shielding, elevated transmittance in visible range, low refractivity, and wide band gap, which are not explored at their full applicative potential [16,17,18]. For instance, tuning the refractive index (n) of HEC by introduction of specific fillers unlocks additional possibilities of using this polymer in fabrication of antireflection coatings for displays, cores for optical fibers, front covers for solar cells, and encapsulants for lighting systems. For such purposes, a trade-off between the high transparency and high refractive index of the composite is necessary for controlling the effects related to the total internal reflection (TIR) phenomenon occurring in such devices. In the case of light emitting diodes (LEDs), a percent of the light generated by the chip remains confined in the device as a result of the discrepancy among the refractive properties at the interface of semiconductor with that of encapsulants (silicon dioxide, acrylates or epoxy resin with n = 1.35–1.50) [19]. This is because the incident radiation that should pass through these layers is stopped due to TIR when the angle of incidence is larger than the critical angle for TIR [19]. To approach this issue, photonic crystal structures [20], omnidirectional reflectors [21], and nano-gratings [22] were used in LED construction. None of these options eliminate the major problem of the refractive index contrast. This is why attention should be given to the optical properties of the encapsulant material. Traditional encapsulants have low refractivity compared to that of III–V phosphides and nitrides, so polymer loading with highly polarizable fillers is a suitable alternative to address this technical issue. It was found that mingling antagonistic elements in the material structure/composition is one of the most efficient approaches to refine the refractivity [23,24,25]. Few studies were conducted on this path, like those reported by Mont et al. [19,26,27], which reveal that when the refractive index of the polymer encapsulant changes, the LED brightness efficiency can be upgraded up to 20%. This can be accomplished, for example, when TiO_2_ particles are introduced in the epoxy matrix to enhance the magnitude of n parameter [27].

A good strategy to increase the refractive index of polymers resides in the incorporation of inorganic fillers, especially metallic ones. The main attention here is focused on filling HEC with a combination of particles of metal oxides and metal salts with the purpose of tuning the refraction behavior of the resulting composites. Among the metal oxides, TiO_2_ is one of the most utilized inorganic fillers that can help to increase the refractive index of polymers because it is characterized by a naturally high refractive index itself, especially in rutile form [28]. Additionally, TiO_2_ has another advantage, namely a thermal conductivity of about 10 W/(m⋅K) [29], which is good for dissipation of heat from devices. This metal oxide is generally regarded as a stable and non-toxic compound, being used as pigment in food [30] or as a UV filter in cosmetic formulations [31]. Micrometer-sized TiO_2_ fillers render opacity and brightness as a result of their larger dimensions, while nano-sized TiO_2_ are more characterized by transparency [30]. There are some concerns about possible toxicity of nano-TiO_2_ via inhalation, but skin contact is regarded as safe [30]. Evans and collaborators [32] studied both micro/nano-TiO_2_ and demonstrated that there are no cytotoxic effects on human B-lymphoblasts and lung cells. When inserting TiO_2_ into a polymer that contains functional groups, this filler may interact with the matrix via hydrogen bonding [33] or electrostatic forces [34]. The processes at the interface influence the composite behavior, particularly in the case of nano-TiO_2_ particles that favor high contact area, rendering materials with advanced properties [35,36]. Thus, the adhesive or repulsive kinds of interactions occurring among the filler and matrix changes the composite performance [36]. Among the metal salts, PbCl_2_ displays intrinsic high refractivity [37], good transparency (depending on the purity of the crystal structure) [38], and high density, which implies smaller volume to achieve a precise filling amount. When mixing such metal salts with polymers, metal ions are incorporated in the matrix and this enhances the value of n parameter [39]. It is important to mention that this substance has undesired effects on living organisms (e.g., irritation, headache, gastrointestinal issues), hence it is not environmentally friendly [40]. In order to prevent toxicity production, this substance must be handled as hazardous waste and kept away from contact with living beings and the environment. Therefore, PbCl_2_ recycling or disposal must be undertaken by following regulations, whereas lab work must be performed with proper equipment that protects them from inhalation or skin contact. Based on the above considerations, these two inorganic fillers were chosen in this work for their intrinsic refractivity, which is helpful to tailor the refractive index of HEC. Moreover, the good thermal conductivity of TiO_2_ is beneficial to dissipate heat from the LED and this was a supplementary selection factor. Also, PbCl_2_ can produce interfacial modification of TiO_2_, influencing its electrical and optical features [41], and this might positively influence the composite properties. As far as we know, the evolution of the optical properties of HEC containing more than one filler has not been reported yet.

From an electrical point of view, HEC can be regarded as a ‘green’ insulator and few reports were focused on its dielectric behavior [18,42,43,44], showing that this polymer presents a low permittivity (under 3) and a broad relaxation process, which is influenced by temperature and moisture absorption. The permittivity is an essential parameter for designing dielectric components for electrostatic energy storage devices, like capacitors. Recently, it was found that cellulose-based dielectrics are quite promising for energy saving [45,46]. Literature studies indicate that the dielectric properties of HEC can be adjusted by reinforcement with salts [47], ammonium thiocyanate [48], copper oxide nanoparticles [14], metal particles [49], and phosphoric acid [50]. These reports reveal that the permittivity of HEC-based materials is affected by the filler features (structure, size, dispersion level), the type of interactions with the polymer, composition, thickness, and ambient conditions (humidity, temperature). In previous studies, we investigated the effect of bio-additives (clays, leaves powder, plant ash) on tuning the dielectric performance of HEC [44,51], opening new perspectives for eco-friendly dielectrics used in energy-saving systems (i.e., capacitors).

Analysis of the described scientific background on specific problems, like encapsulants for LEDs and ‘green’ dielectric for capacitors, leaves open research questions, such as the following: (i) How can we concomitantly improve the transparency and refraction properties of biopolymers like HEC for obtaining LED encapsulants that reduce TIR losses? (ii) Beyond the limit of high transparency (under 80%), are the dielectric properties of HEC-based materials suitable for capacitor uses? The envisioned research in this article attempts to clarify these aspects, so the paper brings innovations compared to existing research by proposing new multiphase composites for LED and capacitor technologies, with advantage of combined flexibility, tailored refraction/dielectric properties, and with a lower impact on the environment, given the biodegradable character of the matrix (the fillers being used in low amounts).

This work continues previous efforts by exploring the applicative potential of novel HEC-based materials. Ternary composites are prepared by loading HEC with a 50:50 mixture of two inorganic and highly polarizable fillers, namely TiO_2_ and PbCl_2_. The variation in the fillers content in HEC on the morphological, optical, and electrical properties is investigated and discussed in regard to the pursued applications. New insights are extracted by inspecting the implications of minimizing optical losses in LEDs via the balance between transparency and refractivity of the attained composites. Also, at high loadings the dielectric properties are properly adapted for energy storage uses.

## 2. Materials and Methods

### 2.1. Basic Materials

The cellulose ether studied here is hydroxyethyl cellulose (HEC) NATROSOL 250, which was purchased under the form of powder from Hercules (Wilmington, NC, USA) and presents a molecular weight of 720 kDa.

Two kinds of inorganic fillers are employed; the first one is Titanium(IV) oxide (TiO_2_) nanoparticles (rutile, <100 nm particle size), and the second one is Lead(II) chloride (PbCl_2_) powder, both acquired from Sigma Aldrich (Saint Louis, MO, USA). The amounts of this metal salt used for sample preparation are very small and this compound was handled with protection equipment.

### 2.2. Composite Preparation

The procedure pursed for the sample preparation is based on the following steps: (a) weighing 0.3 g of HEC powder and mixing with 4 mL of distilled H_2_O; (b) weighing variable amounts of 50:50 mixture of PbCl_2_ and TiO_2_ and introducing in 4 mL of distilled H_2_O for ultrasonication for several minutes; (c) blending the polymer solution with filler dispersion and stirring for 30 min; (d) casting the systems on glass slides, drying at high temperatures (50 °C for 12 h and 60 °C for 12 h), and stripping from the supports. The 50:50 weight ratio of PbCl_2_ and TiO_2_ was chosen to reflect a balanced contribution in terms of costs, optical properties (transparency, refractive index), and thermal conductivity. Final films present an average thickness of 36.6 µm, which was measured with a digital micrometer.

### 2.3. Characterization Techniques

Optical microscopy pictures of the specimens were collected on ADL 601P apparatus (Bresser, Rhede, Germany) in transmission mode.

Atomic force microscopy measurements were conducted using an NTEGRA system supplied by NT-MDT Spectrum Instruments Company, located in Zelenograd, Moscow, Russia. An NSG 10 cantilever (TipsNano OÜ, Tallinn, Estonia) with a resonance frequency of 190 kHz and a normal spring constant of 14.1 N/m was used to examine the surface texture of the samples under ambient conditions at 23 °C. The working procedure was in tapping mode. The program Nova 1.1.1.19891 (NT-MDT Spectrum Instruments, Zelenograd, Moscow, Russia) was used in the process of capturing the images on surfaces varying between 60 × 60 µm^2^ and 1.5 × 1.5 µm^2^ and for morphological analysis. The scanning area of 20 × 20 µm^2^ was selected as representative.

Colorimetry analyses of the studied films were performed on CL-70F apparatus (Konika Minolta, Tokyo, Japan).

UV-VIS spectroscopy data were registered in transmission mode on Jasco V-670 ((Jasco Int. Co., Ltd., Tokyo, Japan) and SPECORD 210 PLUS instruments (Analytik Jena GmbH, Jena, Germany).

Refractometry data of the attained films were achieved on Abbemat setup (Anton Paar GmbH, Ashland, VA, USA) measured at 26 °C.

Dielectric spectroscopy experiments were performed on a Novocontrol Concept 40 instrument (Novocontrol Technologies, Montabaur, Germany) at room temperature and variable frequencies (1–10^6^ Hz).

## 3. Results

A comprehensive analysis of the HEC-based composites is conducted for elucidating the effect of filler mixture on the relevant properties for targeted applications. For instance, composition-tuned optical behavior has great importance in designing encapsulants capable of enhancing the light extraction from LEDs, rendering brighter light sources, while the dielectric properties are relevant for making components for capacitor devices.

### 3.1. Morphological Analyses

**Figure 1:**
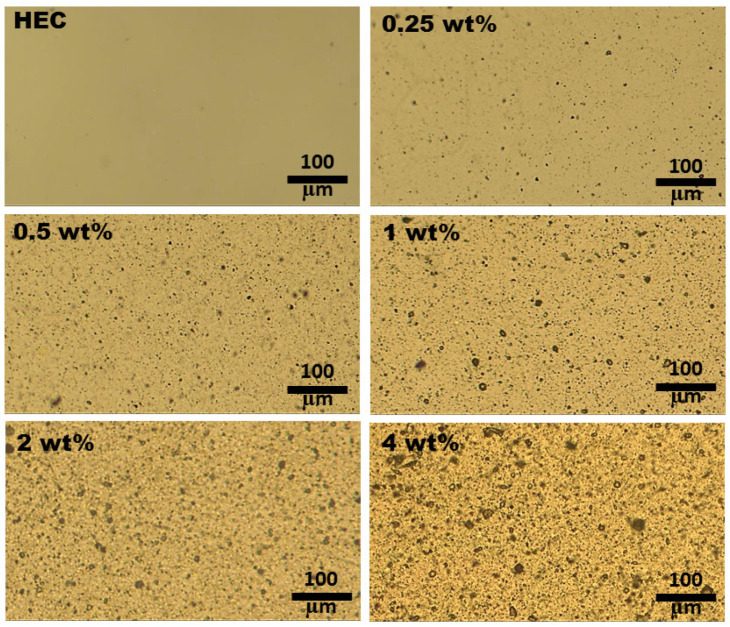
The optical microscopy images of HEC matrix and HEC/TiO_2_+PbCl_2_ composites containing 0.25 wt%, 0.5 wt%, 1 wt%, 2 wt%, and 4 wt% fillers.

The morphological characteristics of the prepared composite films are examined since homogeneity is very important for applications. To observe the morphology at macro-level optical microscopy images were recorded for all specimens (Figure 1). Prior to filling, the HEC film presents a relatively smooth surface and no defects. After the gradual incorporation of the TiO_2_+PbCl_2_ filler mixture in the matrix, the morphology of the samples is modified. The presence of the used fillers is remarked under the form of black dots surrounded by the transparent polymer. At all compositions these dots are well distributed within the cellulosic environment as far as optical micrographs reveal. As the filler content increases from 0.25 wt% to 4 wt%, the dimensions of the dots are higher, but no large agglomerates are formed.

**Figure 2:**
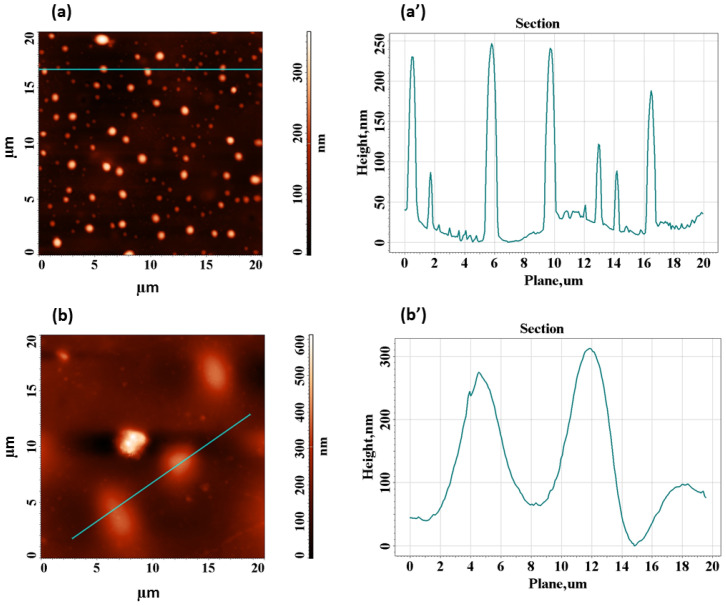
2D-AFM images and the corresponding surface profiles (taken along the line from the corresponding topography images) for HEC/TiO_2_+PbCl_2_ films containing (**a**,**a’**) 0.5 wt% mixture of TiO_2_+PbCl_2_ particles, and (**b**,**b’**) 4 wt% mixture of TiO_2_+PbCl_2_ particles.

Additional analysis of the morphology of the films is undertaken at nanoscale with the help of AFM technique. In a previous work [51], we showed via AFM scans that the neat HEC sample displays a very smooth surface (the largest height of the surface formations not reaching 10 nm). Also, HEC layer presents an average roughness of about 0.5 nm recorded for a scanned surface of 5 µm^2^. The 2D surface topography of the composite films containing 0.5 wt% and 4 wt% TiO_2_+PbCl_2_ fillers is depicted in Figure 2a,b. At the loading level of 0.5 wt%, one may notice in Figure 2a that the particle mixture is well distributed within the cellulosic matrix. The surface of this composite film is characterized by an average roughness of 23.8 nm, across the scanning area of 20 µm^2^. The corresponding surface profile (Figure 2a’) reveals that the height of the surface formations ranges between 150 and 250 nm, while their width is in the interval of 850–1000 nm. At the highest level of loading of 4 wt%, one may distinguish in Figure 2b that the inorganic particles tend to form tiny agglomerates which are distributed in the matrix. Because of this, the average roughness is almost doubled (being of 44.6 nm) along the same scanning area of 20 µm^2^. The corresponding surface profile (Figure 2b’) indicates that the surface heights increase up to 300 nm and the width becomes larger (around 6 µm). The differences in the surface features are induced by the distinctness of the sample’s composition and this might affect the interaction with the light. The literature [52] shows that the roughness is a crucial parameter that might determine light scattering. Rougher materials tend to produce light scattering (diffuse reflection) to a greater extent comparatively with materials with a smoother surface which mainly reflect light in a single angle (specular reflection). The level of scattering is greater when the peak-to-valley height of the surface roughness is similar or higher than the wavelength of incident radiation [52]. Therefore, at lower filling degree of 0.5 wt%, the composite film is more suited for optical uses.

Increasing the roughness and interfacial contact area with a higher content of the mixture of fillers (TiO_2_ and PbCl_2_) in the matrix can favor better adhesion and enhanced mechanical anchoring of the electrodes [53] to create eco-friendly and flexible dielectrics for capacitor devices. Additionally, rising roughness can induce bending reliability, as the micro/nano-relief can relax stresses during flexion. These aspects are advantageous and can contribute to long-term stability and improved performance in such applications, because they lower the risk of delamination during repeated bending cycles and/or repeated thermal cycles.

### 3.2. Colorimetry Tests

**Figure 3:**
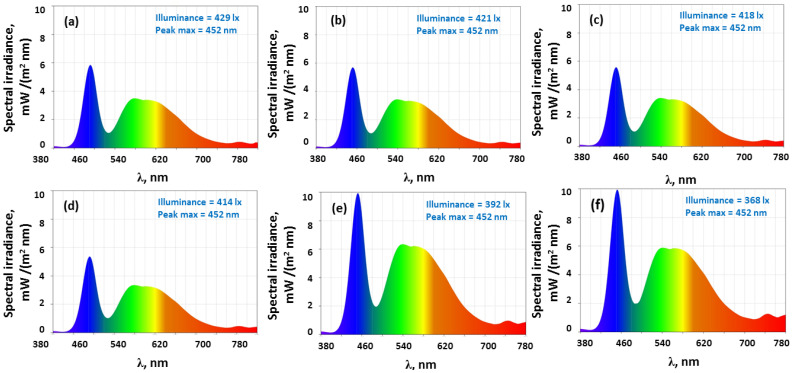
The spectral power distribution of (**a**) neat HEC and HEC/TiO_2_+PbCl_2_ composites containing (**b**) 0.25 wt%, (**c**) 0.5 wt%, (**d**) 1 wt%, (**e**) 2 wt%, and (**f**) 4 wt% fillers.

The examination of color modification upon the reinforcement of HEC with the TiO_2_+PbCl_2_ filler mixture is undertaken on an illuminance color meter. In Figure 3 the spectral power distributions of in the visible domain (380–780 nm) registered for the specimens placed in contact with the device sensor under exposure of a light source are presented. As observed in the registered data, the addition of the fillers in HEC determines the decrease in the values of the illuminance, from 429 lx (neat HEC) to 368 lx (4% TiO_2_+PbCl_2_). Hence, the amount of optical radiation reaching the sample surface per unit area is reduced upon reinforcement. However, regardless of the composition, the specific wavelength at which the source emits radiation through the samples at maximum intensity remains unchanged at 452 nm. So, the presence of fillers in the cellulosic matrix does not disturb the point where the main light energy is concentrated. The amount of radiant energy at 452 nm and around 500–660 nm wavelengths from the visible spectrum is changed especially at 2 wt% and 4 wt% filler mixture in comparison with the pure HEC film. This aspect is highly relevant since it depicts how colors of the samples are perceived under illumination, as different wavelengths are absorbed or reflected. Aside from the spectral power distribution analysis, chromatic characteristics of the samples are also examined for understanding of the color rendering at various compositions.

The tristimulus parameters (X, Y, Z) are determined by the measuring device upon integrating the specimen’s spectral transmittance–properties curve with the color matching functions of the observer and chosen illuminant. The obtained values are listed in Table 1. Using these data, it is possible to evaluate the CIELAB coordinates (L*, a*, b*) as shown by Equations (1)–(3) [54,55]:

L* = 116 ∙ (Y/Y_n_)^1/3^ − 16, (1)

a* = 500 ∙ [(X/X_n_)^1/3^ − (Y/Y_n_)^1/3^], (2)

b* = 200 ∙ [(Y/Y_n_)^1/3^ − (Z/Z_n_)^1/3^], (3)

where L* refers to the lightness parameter (0—black, 100—white), a* refers to the red/green parameter, b* refers to the yellow/blue parameter, and X_n_, Y_n_, Z_n_ are the reference tristimulus data.

As noted in Table 1, the color temperature (T_ct_) of the samples decreases as the loading level is higher. Thus, the composite films exposed to illumination lead to a smaller correlated color temperature, which reveals that the source emits warmer light (changed towards yellowish tones). The results regarding the CIELAB coordinates (L*, a*, b*) are presented in Table 1. Moreover, the values of the lightness factor L* decrease after gradual insertion of the fillers in the matrix. This means that the color of the composites appears darker to the observer. Also, the magnitude of a* parameter changes from small and positive values for the neat polymer (indicating a neutral hue) to negative values after reinforcement with TiO_2_+PbCl_2_ particles (indicating a slight shift towards green color). Conversely, the values of b* parameters are higher upon incorporation of these two fillers, which reveals that the composites have an almost unperceivable tendency to render fade yellow. Therefore, the composition of the samples can be used to tailor the colorimetric parameters. At filling levels up to 0.5 wt% the colorimetric parameters of the samples do not change significantly enough to affect the emitted color fidelity or brightness of the device, especially for LEDs with GaAsP semiconductors that emit in orange/red zone of the visible spectrum.

### 3.3. UV-VIS Spectroscopy Investigations

**Figure 4:**
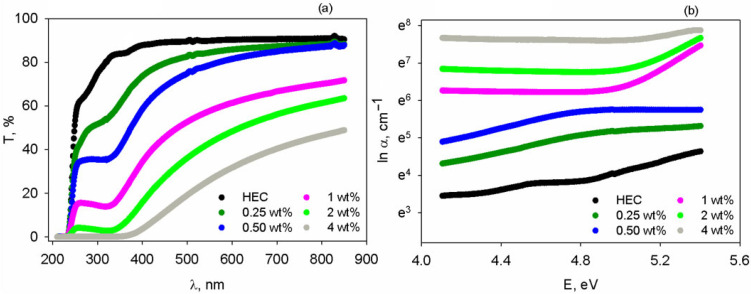
(**a**) Transmittance versus wavelength and (**b**) absorption coefficient versus photon energy of the HEC and HEC/TiO_2_+PbCl_2_ composite films.

The spectral properties of the free-standing films were measured in the 210–850 nm spectral range, as depicted in Figure 4a. The level of light transmission through the neat HEC sample is very high, namely ~80% in the wavelength interval of 320–850 nm. Progressive incorporation of the mixture of TiO_2_+PbCl_2_ fillers produces a slight shift in the absorption edge towards higher wavelengths. Additionally, HEC loading has an impact on reduction in the light transmission level. However, at low loadings of particle mixture (below 1 wt%), one may see that starting with 589 nm the magnitude of the transmittance is high (>80%). Such evolution of optical transparency was found in many polymer composites [56,57,58]. High transparency is advantageous if the samples are used for construction of LEDs emitting yellow/orange/red light.

The spectral results are further used to evaluate absorption coefficient (α), as shown by Tauc’s theory [59] for amorphous materials according to Equation (4):

(4) $$\alpha =\frac{1}{h}ln\left(\frac{1}{T}\right),$$

where h is the film thickness.

The absorption coefficient spectra of the samples are presented Figure 4b, showing that the magnitude of α is enhanced upon filling HEC with TiO_2_+PbCl_2_ particles. Their higher polarizability favors greater possibility of electron displacement under the effect of light, explaining the higher absorption.

At energies under the band gap, the dependence of α = α(E) can follow the Urbach rule [60], shown in Equation (5):

(5) $$ln\;\alpha =ln\alpha_{0}+E/E_{u},$$

where α_0_ is a constant and E_u_ is the Urbach energy.

The E_u_ parameter was assessed from the slope of the semi-logarithmic plots lnα versus E from Figure 4b and the resulting values are included in Table 2. The incorporation of the inorganic particles determines an increased disorder, which renders higher E_u_ values for the composites in comparison with the neat polymer. This is in agreement with other reports on polymer composites [61].

Further, Tauc’s method [59] is utilized to calculate the optical band gap (E_g_), according to Equation (6):

(6) $$\alpha E=C_{0}{\left(E-E_{g}\right)}^{m},$$

where C_0_ is a constant and m refers to the index revealing the nature of the electronic transitions.

**Figure 5:**
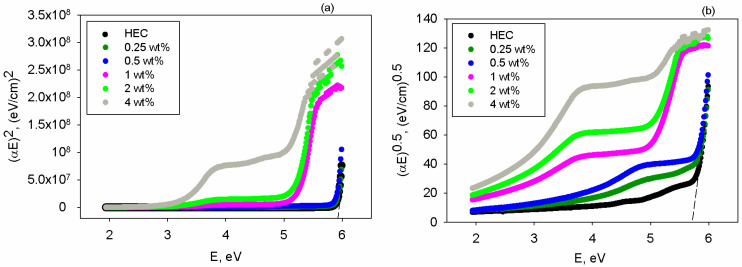
The dependence on photon energy E of (**a**) (αE)^2^ and (**b**) (αE)^0.5^ for HEC and HEC/TiO_2_+PbCl_2_ composite films.

According to the literature [62], m parameter can take the value of 1/2 for direct allowed transitions and 2 for indirect allowed transitions. The plots (αE)^2^ vs. E and (αE)^0.5^ vs. E for the studied materials are shown in Figure 5a,b. By analyzing the linear part of these graphs at the point where an intersection with the *x*-axis is attained, the value of the band gap of each specimen is evaluated. The results concerning the direct (E_g-d_) and indirect band gap (E_g-i_) are shown in Table 2, where one may observe that at all compositions the value of E_g-d_ is higher than that of E_g-i_. The HEC matrix presents a larger band gap, as in the case of transparent polymers [61]. After inserting the mixture of TiO_2_+PbCl_2_ fillers, the diminishment of the band gap magnitude is noted since these polarizable inorganic materials may cause greater electron delocalization and alter the energy levels. According to Table 2, up to 0.5 wt% fillers, the band gap is slightly reduced but remains wide enough to allow transmission of light emitted by the semiconductor (e.g., encapsulant transmittance level is high at 0.5 wt% filler mixture—Figure 4a), preserving LED performance in terms of light output.

### 3.4. Refractometry Investigations

**Figure 6:**
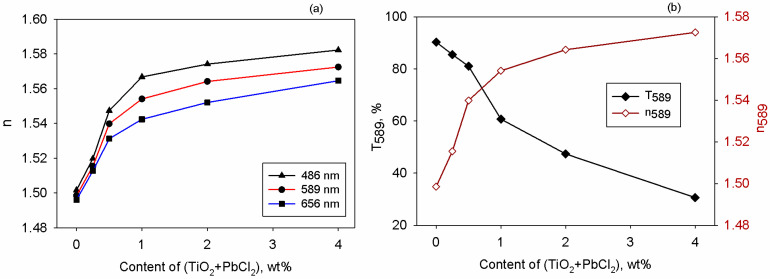
Dependence on the content of filler mixture of (**a**) refractive index measured at three wavelengths and (**b**) transmittance and refractive index at 589 mn for all samples.

The refraction properties of the studied materials were measured at three wavelengths with refractometry which is a suitable method for determining light–sample interaction [63]. According to Figure 6a, regardless of the incident light energy, one may notice that the refractive index increases as the content of the mixed TiO_2_+PbCl_2_ particles is increased. This is also remarked for other polymer composites containing TiO_2_ [64] or inorganic fillers [65,66,67]. The examined films display normal dispersion characteristics (n becomes lower as the wavelength is larger). Analysis of light refraction through the specimens is important for making components used in photonic devices like LEDs because n provides information on light speed and bending when it is passing through distinct device components. For designing proper LED encapsulants, an optimal balance between refractivity and transparency is required. To assess this aspect for the HEC-based, the dependence of n and T measured at 589 nm against the sample’s composition is depicted in Figure 6b, showing that the best trade-off between the optical properties is achieved at 0.5 wt% mixture of fillers in HEC.

In composite materials, the amount of filler inserted in the matrix is known to affect the stability and durability. High filler quantities in the matrix enhance the mechanical properties (strength, stiffness, wear resistance) up to the point where excessive reinforcement produces brittleness [68]. Some negative aspects of high filler contents include the diminishment of transparency, thermal expansion, moisture absorption, and shrinkage. Hence, 0.5 wt% can be regarded as an optimal composition for good flexibility, average hardness, high transparency, and high refractivity, which support the ability to withstand degradation and could expand the lifespan in comparison to the samples at high filler loadings.

For further investigations the HEC/TiO_2_+PbCl_2_ 0.5 wt% sample is considered an encapsulant of an LED with GaAsP semiconductor that emits light starting with 590 nm. Optical features of the GaAsP material have been previously reported [69]. For comparison reasons the same LED with traditional silicon dioxide encapsulant (n = 1.45 [70]) is taken as reference. The thermal stability of traditional SiO_2_ is high since it degrades starting with ~600 °C [71], while for the HEC the onset of thermal degradation is lower, being around 300 °C [72]. However, an encapsulant for LED must endure temperatures varying between 85 °C and 200 °C [73], so the new encapsulant has suitable thermal properties that ensure the device longevity.

**Figure 7:**
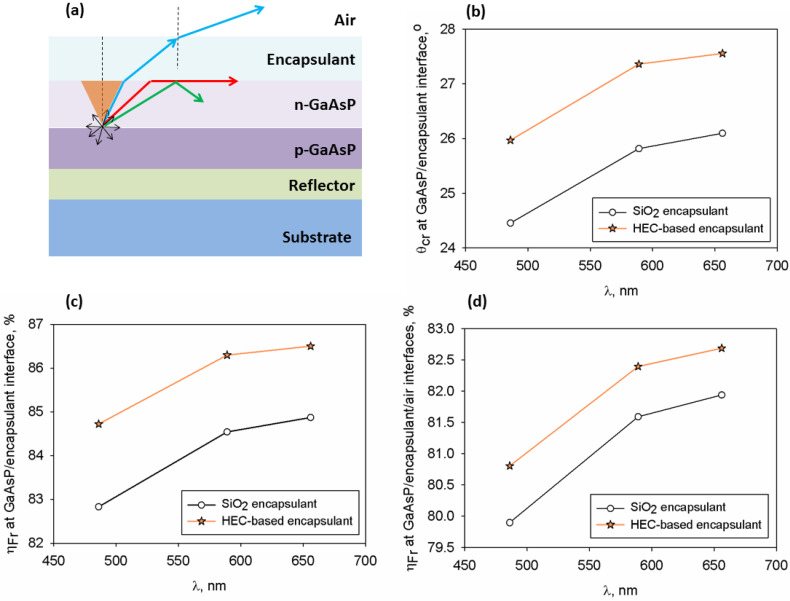
(**a**) Schematic representation of the GaAsP-based LED structure and the light escape cone, (**b**) variation in the critical angle at GaAsP/encapsulant interface with wavelength, (**c**) Fresnel loss factor at semiconductor/encapsulant interface, and (**d**) Fresnel loss factor of light passing via the semiconductor/encapsulant/air interfaces.

The schematic structure of a GaAsP-based LED is displayed in Figure 7a. Since the light extraction efficiency of LEDs is affected by the critical angle (θ_cr_) for total internal reflection and Fresnel loss efficiency (η_Fr_), these factors are next evaluated based on the refraction properties of each of the aforementioned materials from the LED structure.

The critical angle for total internal reflection at the semiconductor/encapsulant interface is determined using the Snell’s law written for normal incidence condition [74]. If the incident light reaches the interface at angles exceeding θ_cr_ then the rays are not entering the encapsulant at all, hence it will be reflected back (leading to TIR). The losses produced by TIR are known as critical angle loss. The θ_cr_ results obtained for both types of encapsulants are presented in Figure 7b. One may see that the higher refractive index of the loaded HEC film is more advantageous for the enhancement of the θ_cr_ in comparison to those obtained for the conventional silicon dioxide encapsulant. For both materials, the magnitude of θ_cr_ increases especially at bigger wavelengths. Based on the critical angle, it is facile to determine the zone where the radiation escapes from the device, and according to the literature a higher critical angle leads to an expanded light escape cone [27]. Thus, the increased refractivity of the composite sample is solving the issue of losses of light generated by the p-n junction of the device.

The light power loss determined by Fresnel reflection loss is also assessed based on refraction properties of neighboring media [75], so η_Fr_ was estimated for the semiconductor/encapsulant and at the semiconductor/encapsulant/air interface, as shown in Figure 7c,d. For both types of materials the values of η_Fr_ are increasing with wavelength. Moreover, one may see that the Fresnel loss efficiency differs as a function of the refraction properties of the encapsulant. Better matching of the refractive index of the prepared HEC encapsulant with that of GaAsP renders greater values of η_Fr_ parameter in comparison with the conventional one. So, the obtained encapsulant diminishes the light losses at the interface with GaAsP layer. The Fresnel loss efficiency of radiation passing via the semiconductor/encapsulant/air media is found to be larger, which is beneficial for the pursued application.

### 3.5. Electrical Properties

**Figure 8:**
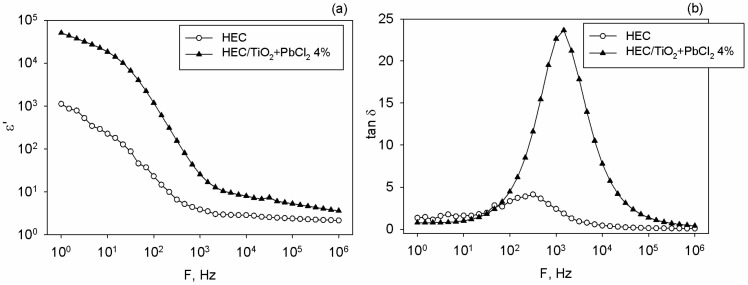
(**a**) Real part of the dielectric constant against frequency, and (**b**) dielectric loss tangent (tan δ) for HEC and HEC/TiO_2_+PbCl_2_ composite films.

The electrical properties of the analyzed films were experimentally determined on a broadband dielectric spectrometer in order to evaluate the frequency-dependent behavior of the real part of the dielectric constant (ε’) and dielectric loss tangent (tan δ). For comparison reasons, the neat HEC and composite at highest loading of 4% TiO_2_+PbCl_2_ were analyzed and the data of ε’ and tan δ against electric field frequency (F) are presented in Figure 8a,b.

Regardless of the presence of the filler mixture, the magnitude of ε’ decreases as the electric field frequency is larger, as noted in Figure 8a. This can be explained by accounting for the delay of the dipole oscillations in regard to the frequency of the applied electric field. The sudden increase in ε’ of neat HEC at low frequency is connected to the electrode polarization effect, which is the result of appearance of electric double layers [76]. When sweeping from low to high frequencies one may observe a change in slope of ε’ curves at an intermediate frequency and this is caused by the dielectric relaxation processes in the HEC matrix. The literature [42] also reveals that this biopolymer displays a broad relaxation that is generated by the reorientation of ethylene oxide moieties or other side groups. In the high-frequency interval, the dipole orientation is slowed down since they are unable to rotate/align under the action of external field, so they are not contributing to polarization and the recorded ε’ is smaller. Similar dielectric behavior is observed for HEC-based composites, and the difference is that the incorporation of the filler mixture renders larger values of ε’, namely at 1 kHz; this parameter is enhanced 6.63 times in regard with the matrix. After 1 kHz the examined films present dielectric stability, i.e., ε’ is less affected by frequency in this interval.

The changes in ε’ upon HEC loading are due to the formation of small domains of inorganic particles which are acting analogously to small capacitors and the distance among them is shortened at higher loadings. However, at high filler loadings, the long-term dielectric stability must be discussed due to potential issues related to filler agglomeration. If particles clump together they can generate localized zones of high electric fields that might determine partial discharges, which ultimately enhances the material’s vulnerability to dielectric breakdown [77]. Agglomerates also enhance the dielectric loss, so under the action of alternating electric field the composite accumulates heat and might degrade [77]. The beneficial influence of inorganic fillers in polymers were previously reported [78,79,80,81,82], including for those containing metallic fillers like Au [83] or TiO_2_ particles [84].

Another important parameter is tan δ, which was measured at several frequencies, as seen in Figure 8b. The pristine HEC presents a peak in tan δ curve around 1 kHz, which might be attributable to the maximum relaxation of the cellulosic chains. At this point, the electric field frequency corresponds to that responsible for the rotation of the molecular segments, so maximum power transfer takes place [85]. After the incorporation of the filler mixture in HEC, the observed peak has a higher intensity and it is moved towards high frequencies. This denotes the reduction in relaxation time as a result of faster charge migration through the HEC-based composite. Thus, the relaxation process within the analyzed material occurs faster than in the case of neat HEC. Such variation in tan δ versus frequency was also reported for other polymer composites [85,86], revealing a type of Debye relaxation. The formation of interfaces among multiple phases, especially in nanocomposites containing ionic counterparts (like PbCl_2_), is responsible for uncompensated charges that under external fields generate space charge effects [83]. The latter can be regarded as extrinsic phenomenon, which at small frequencies might determine the augmenting of the dielectric losses [83].

## 4. Conclusions

The current paper is focused on the preparation of a novel set of ternary polymer composites made of HEC, which is filled with variable amounts (0.25–4 wt%) of a 50:50 mixture of TiO_2_ nanoparticles with PbCl_2_ micropowder. Optical micrographs reveal that the attained composites present an adequate homogeneity at macro-level, with a small tendency of aggregation starting with 1 wt%. Additionally, AFM data support the fact that the surface roughness is modified as a function of added inorganic particles and, at 0.5 wt%, the height of the structural formations is lower than the light wavelength-avoiding scattering phenomenon. Transmittance results support this and show that at the composition interval of 1–4 wt% the samples let a smaller amount of light pass. Colorimetry tests also reveal a decrease in the lightness factor at high loadings of HEC and an almost unperceivable tendency of shifting hues towards fade yellow. Analysis of the light dispersion properties of the specimens points out that the refractive properties decrease at bigger wavelengths and are enhanced upon insertion of the filler mixture. The best balance between transparency and high refractivity is achieved for the composite containing 0.5 wt% fillers. When using this sample as LED encapsulant it was proved that at 589 nm the Fresnel loss efficiency of light traveling via the semiconductor/encapsulant/air is found to be 1.01 times greater than that of the LED encapsulated with conventional silicon dioxide. Furthermore, electrical characterization by broadband dielectric spectroscopy demonstrates that the presence of filler blends in HEC produces the formation of polar domains, leading the 4 wt% to an augmentation of the real part of the dielectric constant by 6.63 times in comparison to the neat HEC. Such results are promising for making eco-friendly dielectric layers for capacitor devices.

## Figures and Tables

**Table 1:** The recorded X, Y, Z—tristimulus parameters, T_ct_—color temperature, calculated L*—lightness, a*—red/green parameter, and b*—yellow/blue parameter.

Fillers, wt%	X	Y	Z	T_ct_	L*	a*	b*
0	412.2346	429.0546	468.3194	6403	97.0355	0.1021	0.6193
0.25	404.4324	421.4169	456.9671	6370	96.3608	−0.0836	1.0386
0.50	401.1617	418.4498	448.3822	6302	96.0964	−0.2532	1.7971
1	394.3654	412.2261	432.4244	6189	95.5379	−0.5884	3.1331
2	375.1916	392.0748	401.9121	6047	93.6900	−0.5349	4.5054
4	351.8888	367.8314	347.6491	5622	91.3809	−0.5689	9.2374

**Table 2:** The values of the film thickness (h), Urbach energy (E_u_), direct band gap (E_g-d_), and indirect band gap (E_g-i_).

Fillers, wt%	h, µm	E_u_, meV	E_g-d_, eV	E_g-i_, eV
0	39	1137.66	5.94	5.73
0.25	35	1184.83	5.87	5.66
0.50	35	1381.22	5.80	5.56
1	37	1605.14	5.31	4.83
2	37	2666.67	5.15	4.65
4	37	4761.91	4.75	3.79

## Data Availability

The original contributions presented in the study are included in the article, further inquiries can be directed to the corresponding author.
